# Thoracic curlicue: A case of ureteral herniation

**DOI:** 10.4103/0970-1591.60462

**Published:** 2010

**Authors:** Sudhir Sukumar, P. Ginil Kumar, Appu Thomas

**Affiliations:** Department of Urology, Amrita Institute of Medical Sciences and Research Centre, Kochi, India

**Keywords:** Ureter, diaphragm, hernia

## Abstract

Isolated herniation of ureter into thoracic cavity due to a defect in the diaphragm is a very rare entity. We present clinical details and radiographic images of an incidentally detected herniation of right ureter into the thorax in an elderly lady with no previous history of trauma or urinary tract symptoms. This acquired but asymptomatic condition is confirmed by radiologic imaging that reveals the classical loop configuration which is described as the curlicue sign. Further evaluation had revealed mild renal failure but with no definite evidence of obstruction. In view of age and asymptomatic status, patient was offered non-surgical management.

## INTRODUCTION

Ureteral herniation is a rare entity of which fewer than 200 cases have been reported, being usually detected incidentally during radiological evaluation or during surgery.[1] They have been described in inguinal, femoral, sciatic, and parailiac locations;[1] occurrence in the thoracic cavity is considered extremely rare.

## CASE REPORT

A 75 year-old lady with long-standing hypertension was detected to have mild renal failure during evaluation for an upper respiratory infection. She did not give any history of colics, previous episodes of urinary tract infections, or trauma to the chest or abdomen. A subsequent abdominal ultrasonogram revealed mild right hydroureteronephrosis with slightly decreased parenchymal thickness.

**Figure 1:**
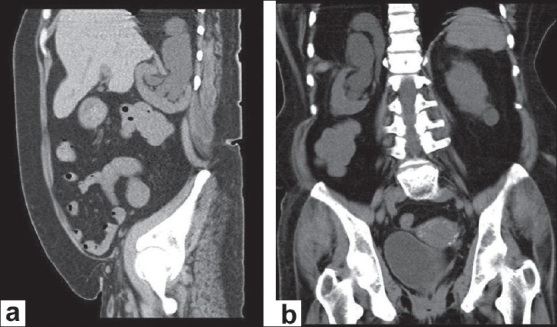
Noncontrast-enhanced computed tomography scan; (a) Saggital images showing malrotated and cranially displaced right kidney with ureter and retroperitoneal fat herniating into thorax; (b) Coronal images showing right ureter looping into thorax and back into retroperitoneum through a diaphragmatic defect

**Figure 2:**
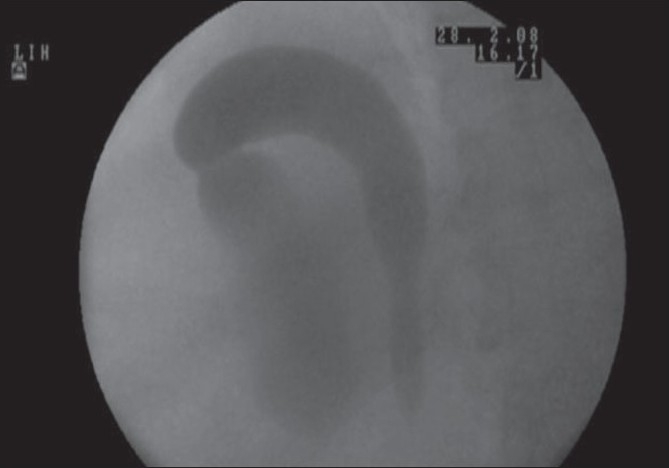
Retrograde ureteropyelogram image showing contrast ascending the right upper ureter in the thoracic cavity and then looping down into the pelvicalyceal system

In view of her altered renal functions, a noncontrast computed tomography scan was done which revealed a relatively small right kidney that was displaced cranially towards the diaphragm; the renal axis was abnormal with the pelvis facing posterosuperiorly [Figure 1a]. Along with retroperitoneal fat, the dilated right ureter could be seen herniating into the ipsilateral thoracic cavity through a 2.6 cm wide defect in the posteromedial aspect of the right hemidiaphragm [Figure 1b], which was suggestive of a Bochdalek hernia. The lower chest sections showed the ascending and descending limbs of the herniated ureter in a loop configuration [Figure 1b]. The descending ureter appeared to be compressed at the location of the diaphragmatic defect. A subsequent retrograde pyelogram confirmed the classical ureteral loop or knuckle, which has been described as the curlicue sign [Figure 2]. It also demonstrated prompt and complete emptying of the pelvicalyceal system, thus ruling out an obstructive cause for the renal failure. Hence, it was decided to manage the patient's renal failure with medical measures. Future surgical intervention has been planned only if there are chest symptoms due to the mass effect, or if renal failure can be definitely attributed to the anatomical anomaly.

## DISCUSSION

Isolated ureteral herniation through a diaphragmatic defect has been sparingly reported in literature;[2,3] these previous reports have also been incidentally detected during either surgery or radiological imaging. Although rare, these are believed to be acquired and the incidence increases with age, being common in those with emphysematous chests. [4] A preponderance has been observed for the female sex[4] and the right side.[1]

Our patient did not give any history favoring a traumatic etiology for the diaphragmatic defect, and hence, may also be assumed to have acquired it due to age-related degeneration. The defect is thought to occur at the junction between the crus and the posterior diaphragmatic fibres.[4]

The diagnosis of ureteral hernia at any site can be confirmed on radiological imaging by the curlicue sign that occurs due to a ureteral loop or spiral.[5] This classical appearance can be demonstrated in erect and oblique projections of roentgenographic images during the excretory phase. [1,5] They are also evident in coronal and axial computed tomographic imaging.[2–5] The herniation may be gradually progressive, involving long segments of the ureter, but may not always require surgical intervention.[2] However, persistence can lead to ureteral obstruction, colics, and subsequent compromise of renal function, besides chest discomfort and respiratory distress in large defects.

## CONCLUSION

Ureteral hernias into the thoracic cavity are extremely rare but are easily diagnosed by the sign of the curlicue ureter. They are usually asymptomatic but early diagnosis may be beneficial, especially in a setting of renal failure.
